# Dr. Granville on the Internal Use of Prussic Acid

**Published:** 1821-03-01

**Authors:** 


### XIII.

*A Practical Treatise on the Internal Use of Hydro-cyanic (Prussic) Acid, in Pulmonary Consumption and other Diseases of the Chest, as well as in several Complaints attended by great nervous irritation, or acute pain, &c. &c.* By A. B. Granville, M.D. F.R.S. F.L.S. M.R.I. &c. &c. Small octavo, pp. 417. *Second Edition*, greatly enlarged. London, 1820.

We noticed the first edition of this work (though not very fully) in our quarterly series, No. 6, October 1819; but the present edition is so much enlarged as to be "a new work, and the fourth attempt which the author has made, in the course of five years, to establish the claims of a new and powerful remedy to the attention of the medical profession in this country." We remember the time when the sanguine hopes held out by this new remedy, would have caused our hearts to palpitate with joy, at the prospect in which suffering humanity might reasonably indulge; but twenty-five years of observation have somewhat chilled our feelings on these occasions, and we have long been convinced that a careful study of the causes, seat, nature, and indications of a disease, will enable the attentive practitioner to effect most of what can be effected, by even a very few of the common remedies now in use. Yet, as long as man is subject to irremediable disease, and consequently death, he will seek after "new and powerful remedies;" and although he will be, myriads of times, disappointed, he will occasionally discover very valuable auxiliaries to the healing art. That prussic acid is a powerful medicine we have no doubt; that it is capable of useful application, in many disorders, we also believe;—but that it will considerably disappoint the expectations of some of its employers, we confidently predict —and that, because more virtues are attributed to it than can reasonably be hoped from any one remedy. But to proceed to our analytical labours.

We shall pass over the chemical history of prussic acid, and also the methods of procuring it employed by Scheele, Vauquelin, and Majendie, in order to give that resorted to by the Apothecaries' Company. The formula was supplied by Professor Brande, and is as follows:—

Prussiate of mercury, ℥j.; muriatic acid, ℥j.; water, ℔v. Draw off four pints, and rectify through chalk. Dr. Granville makes some objections to this process, observing that the acid cannot thus be obtained in a uniform and well-known state of concentration.*

---

* Since this analysis was drawn up, some observations on these objections to the Company's process, have appeared in the Journal of Science, wherein it is confidently stated that the process employed at the Hall, is, upon the whole, less objectionable than any of the others; and that the prussic acid thereby obtained, is more uniform in its strength, and better adapted to medicinal purposes.

In Dr. Ure's very excellent Dictionary of Chemistry, recently published, and which we strongly recommend to our brethren, we perceive the following "ingenious and accurate process for preparing prussic acid for medicinal purposes," communicated to Professor Ure by Dr. Nimmo, of Glasgow:—

"Take of the ferroprussiate of potash 100 grains, of the protosul-

In the fourth section our author details the physical properties of prussic acid, especially when prepared according to Gay Lussac's method.

"1. At the common temperature it is liquid, colourless, and transparent, with a strong smell of bitter almonds, and a peculiar pungent bitter taste, at first bland and sweetish.

"2. It is volatilized at 20° of the centigrade thermometer, boils at 26°, and at 15° below 0 it becomes concrete, and crystalizes in needles like nitrate of ammonia. Its extreme volatility is such, that when a drop of it is exposed to the air, on the end of a glass rod, it is rapidly crystalized. The same happens if a drop be suffered to fall on a sheet of paper.

"3. Its specific gravity is 0.70583: when in a concrete state it is only 0.600: while that of its vapour is 0.947.

"4. It has an odour so strong and characteristic, that it produces almost immediate pain in the head, with deafness, unless *largely* diluted with air or water, as in the case of the acid prepared for medicinal purposes. In other respects the smell is the same as that of peach flowers or of bitter almonds.

"5. Its tendency to assume the gazeous form is very considerable: it is decomposed by a high temperature, or by the contact of light, when carbonic acid, volatile alkali, and carburetted hydrogen gas are given out, a carbonaceous matter remaining behind.

"6. When brought near to a body in a state of combustion, it instantly inflames and burns with a blue flame. Water and alcohol dissolve it readily." 30.

The 5th section is devoted to the properties of this substance on the vital machine, as ascertained by physiological experiments. The principal of these are, that when administered, in its concentrated state, to warm blooded animals, it destroys their sensibility, and the contractility of their voluntary muscles.

---

phate of iron 84½ grains, dissolve them separately in four ounces of water, and mingle them. After allowing the precipitate of the protoprussiate of iron to settle, pour off the clear part, and add water to wash the sulphate of potash completely away. To the protoprussiate of iron, mixed with four ounces of pure water, add 135 grains of the peroxide of mercury, and boil the whole till the oxide is dissolved. With the above proportions of peroxide of mercury, the protoprussiate of iron is completely decomposed. The vessel being kept warm, the oxide of iron will fall to the bottom, the clear part may be poured off to be filtered through paper, taking care to keep the funnel covered, so that crystals may not form in it by refrigeration. The residuum may be treated with more water, and thrown upon the filter, upon which warm water ought to be poured, until all the soluble part is washed away. By evaporation, and subsequent rest in a cool place, 145 grains of crystals of the prusside or cyanide of mercury will be procured in quadrangular prisms." *Professor Ure's Dictionary of Chemistry; art. Hydro-cyanic Acid.*

"But even the strongest hydro-cyanic acid of Gay Lussac, though dangerous in its preparation, soon ceases to be so, as it is impossible to preserve it in a state of purity more than a few hours, a fact to which we have already alluded.*" 44.

This seems a great objection to the general use of the remedy, especially in the country, where it is not likely to be often prepared.

In respect to the action of prussic acid on the nervous system, our author cannot say whether it is directly sedative, or first excitive, followed quickly by a sedative operation. The same question has been long agitated respecting the properties of digitalis. It is, however, as Dr. Granville properly observes, quite sufficient for practical purposes, to ascertain the aggregate sum of all the visible effects which the prussic acid produces. If the picture of its effects† be not overcharged, we are possessed of a valuable Nepenthe indeed.

Dr. Granville here enumerates cursorily the various diseases to which this remedy is applicable; for which list see page 271 of the second volume of our quarterly series.

Since the first edition was published, Dr. G, has administered the prussic acid, as a palliative, in organic, or incurable diseases, and he seems to think with much good effect.

The 8th section, on the poisonous properties of this medicine, when taken in too large doses, we must pass over, though containing much curious and interesting matter. The 9th section also, containing a history of the introduction of the prussic acid into medical practice, does not come within the scope of our analysis.

Our business lies most with the second part of our author's work, containing "a short preliminary account and delineation of the various complaints in which the prussic acid has been administered, as a medicine." In the first section of this part, Dr. Granville fires some tremendous broadsides, right and left, against certain critical benches in this metropolis. But as we are, happily, not included in the number, we shall leave our brethren on the benches to fight their own battles.

In those cases of pulmonary consumption, depending on hereditary organization of the chest and respiratory apparatus, our author has exhibited the acid—not with the hope of

---

* Gay Lussac says, "En conservant cet acide dans des vases bien fermés, même sans qu'il ait le contact de l'air, il se décompose quelquefois en moins d'une heure." Op. citat.

† See page 270 of vol. ii. quarterly series.

preventing the fatal process going on; "but with a view to allay the nervous irritability, restlessness, and watchfulness of the patient." In this, however, Dr. G. was disappointed; for the prussic acid proved equally unavailing as opium, hyoscyamus, digitalis, lactucarium, &c.

Tubercular and strumous phthisis, Dr. G. takes an opportunity of remarking, may probably be often owing, in this country, to the too great quantities of butter given to children. In what is known by the first stage of this species of phthisis—that is, before any suppuration or softening down of the tubercles, Dr. G. thinks, if the prussic acid be given, "a perfect recovery may be expected." Here our author relates several cases in his own and other physicians' practice, illustrative of this statement; but we confess that the cases have not left that impression on our minds, which they are meant to convey. Thus in the first case, (Majendie's) no symptoms whatever are detailed, and it is only stated generally that the young lady had—"every symptom of incipient consumption." In the second and third cases (Dr. Scudamore's) the patients had "*puriform* expectoration," which entitles them to be placed beyond the range of the *incipient* stage; and if the expectoration were really pus, it is too much to think that they could have been so easily cured by the prussic acid. In the fourth case (Master Blackwell) Dr. Granville himself gave in an unfavourable prognosis to Sir Walter Farquhar. "The boy was wasting daily—the cough and night sweats had manifested themselves in a decided manner." He took the prussic acid, and, "in about three days the disorder seemed arrested." In a fortnight he was well, with the exception of debility. We put it to the experience of our author, and of our brethren at large, whether such sudden cures are at all compatible with what is designated tubercular consumption, in any stage whatever, and particularly where daily wasting and other formidable symptoms have established themselves.

In the tenth case we are at a loss to conceive how Professor Brera could consider it in the *first* or *inflammatory* stage, when he concludes thus:—"nam sputorum quantitas decrevit, eorumque *purulentia* in melius se commutavit; quæ originem ex *tuberculis* ducebat *suppuratis*, antequam æger clinicum institutum subiret." 163. If then the Italian Professor cured a patient with *suppurated tubercles*, there is no longer any question about the power of prussic acid over *confirmed* consumption.

In the next section Dr. Granville states, and we think very truly, that, "when the *suppurative* stage of the tubercles has fairly begun, the hopes of recovery from the effects of the prussic acid, become every day more faint, &c." Still, however, our author thinks that, not only alleviation of symptoms, but even recovery, in some instances, may be looked for, from the remedy, and "that even at a very advanced period of this complaint." Dr. G. supports this hope from what has resulted from *other* medicines, "under the most unfavourable circumstances," "as Dr. Lænnec has proved." But if our readers will consult Lænnec, or our analysis of his work, at page 467 of our last volume, they will find that these cures in suppurated tubercles, resulted from a spontaneous process of Nature, namely, a "transformation of the pulmonary ulcerations into semi-cartilaginous fistulæ," and consequently, not from any particular remedy. Now these observations are not meant to discourage the use of prussic acid; but to moderate the too sanguine expectations of the prescriber, lest a valuable medicine be brought into discredit, from an exaggerated estimate of its powers.

We must pass over a great mass of cases of Dr. Granville's and his correspondents, which we cannot analyze, and therefore on which we shall not give an opinion.

In the second section, our author endeavours to shew that, in pneumonia and other inflammatory complaints, after we have carried vascular depletion as far as prudence will permit, the disease is still not quite subdued, and at this juncture, he thinks, "the prussic acid will be found productive of the most advantageous effects." We wish that further experience may confirm this, and also that the colchicum may support the high character lately given of it, in similar cases.

At page 304 we were a little surprized at the boldness of the following assertions.

"I will, however, state, once for all, that the whooping-cough, in itself, is *never* an inflammatory disease—for no traces of inflammation have been found in the respiratory organs of those who have fallen victims to it—and that when the complaint has been very violent, and has lasted a great length of time; and then only tokens of inflammation have been found in the brain, as the result of strong and often repeated spasms of the organs of respiration, producing a great determination of blood to the head." 305.

Surely Dr. Granville will not weigh the nosological opinion of Cullen against the evidence of dissection. Dr. Watt, of Glasgow, has demonstrated inflammation of the mucous membrane of the lungs in six cases of fatal hooping cough, three of them, unfortunately, his own children. Were the evidence of an obscure anonymous reviewer of any weight, we might assert that we have opened several children, and one very lately, where the most decided marks of pulmonic inflammation were shewn on dissection, after hooping cough. It is true that our author quotes a passage from an Italian work to shew that in twenty-three anatomical investigations of children after death from hooping cough, there was cerebral turgescence or effusion in all—and "that no symptoms of disease occurred in the chest, *except in a few individuals, who presented an incipient phlogosis or plethora, or serous effusion in that cavity*." With all due deference for our foreign brethren, we beg leave to attach at least as much credit to the statements of our own countrymen, who have almost unanimously agreed, that whenever hooping cough presents febrile symptoms, which, in most of the severe cases it does, then there is bronchial inflammation. In respect to hooping cough producing great debility from the non-oxygenation or non-decarbonization of the blood, this phenomenon was distinctly stated seven years ago, in a former series of this Journal, while reviewing the work of Dr. Watt.

"But may not," says the reviewer, "the dark and singular condition of the blood be attributed to the conjoined influence of disordered respiration, and of the morbid state of the mucous membrane, rendered further impervious by effusion into the air cells and bronchiæ, if such effusion had, at that period, taken place? Thus would the access of atmospheric air to the blood of the pulmonic vessels be greatly obstructed; and may we not, on this principle, explain the sudden and extreme prostration of strength, which occurred in this case; and which characterizes, more or less, the other diseases of this important membrane." *New Med. and Phys. Journ. for February*, 1814. P. 165.

Dr. Watt imagined that in hooping cough there may exist —"some eruptive disease of the membrane of the air-cells and bronchiæ, so minute as to escape ordinary observation, yet so considerable as to excite that inflammation," which seems principally to constitute the disease. This, of course, is ingenious conjecture, not confirmed by observation. Our own opinion is, that the specific contagion of hooping cough is applied to, and has its proper domicile in, the mucous membrane of the lungs, where it excites irritation and spasmodic cough, very generally accompanied by more or less of specific inflammation in the aforesaid membrane. That prussic acid is a valuable remedy in this affection, notwithstanding Dr. Elliotson's unfavourable report, we have no doubt; and we recommend to the perusal of our brethren the numerous cases detailed from page 305 to page 319, as affording very presumptive evidence at least on this point.

At page 320, Dr. Granville enters on the consideration of asthma, and the good effects of the prussic acid in the spasmodic species of this disease. When we say *spasmodic*

asthma, we must beg the forbearance of those gloomy pathologists, both in France and England, who see nothing but *organic* disease of the heart in every case of asthma. That the functions, and ultimately the structure of the heart, occasionally suffer in severe and long-continued spasmodic asthma, especially as life advances, no one can reasonably doubt; and this is the opinion entertained by the most enlightened pathologists of that capital where M. Rostan resides. Some of our countrymen, however, are so captivated with the organic theory of the French pathologist, that they have adopted it apparently without reflecting on the influence which the *nervous system* exerts on respiration, and the symptomatic disorders of the latter from derangements of that system. If M. Rostan has found organic disease of the heart in the dissection of asthmatics, others—many others, equally worthy of credit, have found no organic disease in any viscus. We refer the reader to the temperate and philosophic treatise of our own countryman, Dr. Bree, especially to the 6th section of the first part, where we only perceive one or two instances of disease of the heart among the numerous examinations of asthmatic bodies collected from the best ancient and modern authors. The very fact that asthmatics, when free from other diseases, generally live to an old age, which is any thing but the case in organic diseases of the heart, is a proof sufficiently convincing that genuine asthma has nothing to do with such organic causes. The truth we believe to be, as Lullier-Winslow has remarked, that the dyspnœa accompanying those melancholy and incurable diseases under consideration, has been confounded with spasmodic asthma, and thus given rise to the doctrine in question.

"L'asthme, tel que nous l'avons depeint, est plus rare qu'on ne pense. Beaucoup d'auteurs ont confondu cette maladie avec la dyspnée, qui n'est autre chose qu'une difficulté de respirer plus ou moins intense, et toujours *symptomatique d'une autre affection, telle que d'une maladie organique du cœur* ou de gros vaisseaux, &c."

The above appears to us to be the true state of the case, after all the disputes between M. Rostan and his opponents.

But to return from this digression, which, it is to be hoped, has not been quite out of place. Dr. Granville administered, with benefit, the prussic acid in the case of Mr. K——, who had suffered from asthma for upwards of six years, his complaint assuming an alarming type, the breathing becoming exceedingly difficult, and being rendered more so by the least exertion, or by atmospherical vicissitudes.

"This state of exquisite irritability—this inexpressible anguish, and all the sufferings it produces, were removed by the free but cautious exhibition of the diluted hydro-cyanic acid. Mr. K—— now coughs but seldom, sleeps well at night, becomes more inured to the change of weather, can easily bear exercise, and ascends a staircase without being forced to stop, or without any oppression at the chest." 322.

Mr. Rudland has communicated two cases to Dr. Granville, the first of which is pretty minutely detailed, and of this we shall present an outline to our readers.

*Case.* Sarah R——, ætat 42, had been, for many years, subject to dyspnœa, habitual cough, and occasional attacks of spasmodic asthma. During the last eighteen months, she had been repeatedly under Mr. Rudland's care, and opium, æther, and other powerful antispasmodics, had been administered, without effect. On the evening of the 26th October, 1819, Mr. R. saw her in a dreadful paroxysm of spasmodic asthma. Venesection had formerly been tried at the commencements of these attacks, without evident advantage. The following prescription was therefore ordered :—

℞. Acidi hydrocyanici ♏xij.

Misturæ amygdalæ ℥vj.

Syrupi tolutani - ʒiij.

Fiat mistura cujus capiat partem quartam secunda quaque hora.

The patient felt something easier soon after taking the first dose, and still more so after the second. "Before taking the third dose the most alarming symptoms were greatly relieved; her respiration became freer, her pulse more regular, her countenance indicating a considerable alleviation of her suffering." She slept several hours this night, and, towards morning, was able to take the horizontal position. Mr. Rudland remarks, that after former paroxysms, great difficulty of breathing, incessant cough, and copious expectoration, generally remained for weeks, and sometimes continued until a fresh paroxysm. In this instance, however, the acid appeared to have removed the morbid spasmodic action before exhalation or effusion had taken place.

In the seventh section Dr. Granville treats of hæmorrhages, painful menstruation, and abortion. Dr. Granville here alludes to the scepticism of Dr. Elliotson respecting the power of controlling hæmorrhage, and brings forward cases from the practice of others and of himself, in favour of the medicine. We shall present an outline of the following case from the American Medical Recorder :—

*Case.* "A man, 25 years of age, of a strong constitution, robust, and of a choleric temperament; subject from his infancy to bleeding from the nose, and latterly to hæmorrhoids, complained for about a year of a pain in the epigastric region, accompanied with a strong pulsation in that part. In the mean time his complaint did not prevent him from attending to his trade. One day after having travelled about to various places in a rough vehicle, he experienced a considerable augmentation of pain, which was followed by a copious vomiting of black blood, and a great degree of debility. Returned home, he complained of anxiety—his countenance expressed but little animation; the heat of the body was increased; his pulse was hard and full, and his bowels were constipated. We gave him a decoction of tamarinds; but he was not able to retain the medicine. The *irritability* of the stomach increased to such a degree, that he rejected every thing that he took, even a solution of gum-arabic. We were obliged to have recourse to glysters, and cold applications over the stomach; despite of all, the vomiting continued, becoming more and more copious, reducing the patient astonishingly. The pulse became small and feeble, his extremities were cold, and the constipation very obstinate. In this state of things, we determined to use the laurel water in the dose of sixty drops in a drink, of which a certain quantity was given every two hours, and at last every hour. It was the only medicine the patient could retain. It merits in this case, the eulogium which Thilenius has given it. The first doses sensibly relieved the patient. The vomiting returned but once a day, in very small quantity, and finally ceased; as also did the pain in the epigastric region, and the anxiety. The patient was in a short time restored, and continues in good health to this day." 332.

Dr. Granville next relates the case of the Hon. Mrs. ——, who had been subject for some time to morning hæmorrhage from the lungs, for which the sulphuric acid, bark, and various astringent medicines had been prescribed in vain. Dr. G. exhibited two drops of the prussic acid every three hours, in a camphorated mixture, and soon checked this complaint.

The case of Ann Dunn, a dispensary patient, is stated at page 333 of the volume. This woman, after weaning her child (during the suckling of which the catameniæ had continued), became affected with uterine hæmorrhage, to an alarming extent, which lasted about ten weeks, accompanied by a severe shooting pain in the breast. Our author first prescribed infusion of roses with sulphuric acid and tincture of rhatany, which relieved the other symptoms, the uterine hæmorrhage continuing. He therefore prescribed the following mixture:—

℞. Infusi cascarillæ - ℥vj.

Acidi hydrocyanici ♏xij.

ft. mist. capiat coch. ij. majora quater quotidie.

In about a week the flooding had completely ceased. Two or three other interesting cases of uterine hæmorrhage are stated between page 336, and page 345, for the particulars of which, we must refer to the work itself.

In the 8th section our author comes to the consideration of nervous diseases, and affections of the stomach. As far as regards nervous diseases our author's statements are not very satisfactory—indeed he does not appear to lay much stress on this part of the section. He alludes to the publication of Dr. Elliotson, reviewed in another part of this Number, as forming a suitable appendix to what was already known on the subject of prussic acid in stomach affections, as recorded by Thilenius, Sprengel, and more recently by Mr. A. T. Thomson. We shall, therefore, here present the analysis of a recent communication from the last-mentioned gentleman to our author, containing "his further experience, with respect to the internal use of the hydro-cyanic acid in different complaints, since the former edition of this work."

Mr. Thomson's further experience, he observes, has confirmed his opinion of the efficacy of this acid in spasmodic coughs; but in hooping cough he has not given it that trial which can authorize a decided opinion of its powers. This acid *relieved* a violent hiccup, supervening on an attack of hæmoptysis, and resisting every other means, but could not be continued, in consequence of the debility it occasioned. In a case of tic douloureux the internal and external use of the acid was unsuccessful.

"It may not be uninteresting," says Mr. Thomson, "to the profession, to know the remedy which finally subdued this very violent attack of tic. The patient was a young lady, under fifteen years of age, of a sanguine temperament and quick parts. She had suffered an attack of the disease about a year before, and was not relieved for many months, although it was less severe than that for which I was requested to see her. The pain, which was most excruciating, and returned every three or four minutes, was situated under the chin, about an inch backwards from the symphysis of the lower jaw; and apparently in the course of the branch of the ninth pair of nerves that supplies the genio-hyoideus muscle. A small knot or hardness could be felt externally, and this was enlarged during every paroxysm. The throat was partially affected, and deglutition somewhat impeded, as well as speech. The screams of the patient, when the paroxysms returned, and the writhings which the torture of the pain occasioned, were most heart-rending to those who witnessed them.

"Finding that every remedy with which I was acquainted had failed, I resolved to try the effect of a powerful mental impression; and, with this in view, made enquiry of the lady, under whose charge she was at school, if she knew of any strong antipathies of her pupil. She informed me she had an unconquerable dislike to a dog which was in the house; and having obtained this information, and acquainted the governess with my intention, I proceeded to the room of my little patient, and informed her with as much gravity of countenance as I could command, that the only other remedy I had in store for her disease, was one which I meant should instantly be resorted to; and that it consisted in rubbing the affected part with the back of the dog. The effect was most extraordinary—her face became as palid as that of a corpse, large drops of sweat formed on the forehead, and the girl appeared passing into the most alarming syncope. She, however, gradually recovered; and from that moment, no other paroxysm of the tic was experienced until eighteen months afterwards, when the disease was arrested on the second day of its attack, by suddenly taking her out of bed, and hurrying her into a shower bath. The reason the dog was not again had recourse to was, that she had very imprudently been informed of the motive which had induced me to propose him to be employed in the former instance. I cannot attempt to give the rationale of this practice; but I leave the facts in the hands of the profession." 376.

In some cutaneous diseases, particularly *acne indurata, and rosacea*, Mr. T. has found the acid extremely useful as an external application.

"I have not given it a fair trial in phthisis, for the result of the few cases in which I have employed it, were not such as would induce me to place much confidence in its remedial powers in that disease, when it is at all advanced. I have seen enough of it, however, to be satisfied that it may do much good if the opportunity occurs of giving it in the early stage of the disease, even when there is a predisposition to its tubercular form." 377.

Mr. Thomson has had many opportunities of ascertaining the powers of this medicine in relieving those affections of the stomach in which alkalies and bitters are usually prescribed; and he is now inclined to ascribe the benefit which sometimes follows the use of alkaline medicines in these complaints, to the well-known power they possess, "of diminishing morbid irritability when applied to secreting surfaces, thereby *enabling* the juices of the organ to be more slowly secreted, and consequently of a more healthy character," rather than to their chemical property of neutralizing the superabundant acid which is always generated in dyspepsia." 386.

Here Mr. Thomson remonstrates with Dr. Elliotson for overlooking his observations on the use of prussic acid in *stomach* affections, published in the first edition of Dr. Granville's work; and consequently claims priority, in its application to such class of complaints, as far as Dr. E. and himself are concerned. Mr. T. thinks it unnecessary to detail more than one case in this class of afflictions, as they were all treated nearly in the same manner, except that purgatives were employed more in some than in other instances.

"Mrs. S——, the wife of the Rev. Mr. S——, æt. twenty-five years, of a delicate, slender habit, the mother of three children, had long suffered from dyspepsia. The chief symptoms in her case were,—a sense of fulness of the præcordia, which impeded respiration, and brought on palpitation of the heart to a distressing degree; flushing of the face, acid eructations, chopping and soreness of the tongue, and continued thirst. Her spirits were also occasionally much depressed, and the least fatigue produced a disposition to fainting. As she had been long under medical treatment, and had taken all the ordinary remedies for such symptoms, without any permanent benefit, I resolved to try the powers of the Hydro-cyanic Acid, having recently witnessed its beneficial effects in the case of Mr. R——, and therefore on the 28th of January, 1819, she commenced the use of the following draught.

"℞. Acidi Prussici ♏j.

Infusi Cascarillæ f℥xj.

Syrupi Croci f℥j.

M. ut fiat haustus bis quotidie sumendus.

"In a very few days she was much better: to use her own expressions, she was less nervous, could take more exercise, and had scarcely had the palpitation since the second day after she commenced taking the draught. The dose of the acid was now increased to two minims, and, in this dose, it was continued until the 1st of March; when the patient, finding she was better than she had been for many years, was directed to leave off all medicine, and to go into the country to recover her strength." 389.

Mr. Thomson concludes by stating it as his opinion, that the prussic acid is "a most powerful and direct sedative, and cannot fail of proving, in the hands of judicious and scientific practitioners, a remedy of the greatest value." 389.

After the body of Dr. Granville's work was worked off, the particulars of a case of consumption, cured by prussic acid, were communicated to him by Mr. Plumbe, of Great Russel Street; and a similar case occurred in his own practice.

The outline of Mr. Plumbe's case was as follows: A woman, aged 30, a nurse in a family of respectability, "had been labouring under symptoms of incipient phthisis, since May last." (Oct. 1820) Digitalis, squills, and various remedies had been used without benefit.

"On the 18th of July, when the Hydro-cyanic Acid was first prescribed, the state of exhaustion of the patient, with the very copious expectoration of pus, seemed to warrant the opinion that she could not long survive." 392.

The patient's cough was now very distressing—her nights sleepless—the perspirations copious—the strength greatly exhausted—"the quantity of pure pus expectorated, about four or five ounces in the twenty-four hours."

"In this state of things, the acid was prescribed by Dr. Scudamore, according to the following formula.

"℞. Acidi Hydro-cyanici m vj.

Decoct. Camphoræ.

Mist. Amygdalæ āā f ℥ iij.

Gutt. nigr. m. xij.

Syrupi Simp. f ʒj.

Capt. 4tam. partem 6tis. horis. 393."

The quantity of acid was afterwards increased two minims every twenty-four hours. In about a week she began to sleep sound; does not expectorate above half the former quantity of pus—her appetite became improved, and her strength increased. "The patient continued to take the acid with the daily increase of two minims, in combination with bark, black drop, &c. as before." In three days Mr. Plumbe found a beneficial change, still more surprizing than before. In short, in a month she was well enough to proceed into the country, "with no symptom of her former disease, but some remains of debility." 395.

As the black drop, bark, and other medicines were employed here *along with* the prussic acid, it is impossible to say, decidedly, that the cure resulted from the latter medicine alone. This much, however, we may venture to say;—that the event proved the purulent expectoration to have come from an unbroken surface; for had it come from ulcerated or broken-down tubercles, the issue would have been very different.

Dr. Granville's own case, Miss Munn, detailed from page 396 to page 400, is a very strong one; and were we less sceptical in respect to the curability of confirmed consumption, we should be staggered in the opinions long impressed on our minds from melancholy experience. We remember that some twenty years ago, we were buoyed up with the most sanguine hopes of curing phthisis, in all its stages, by digitalis; the testimonies in favour of which were then far stronger than those of prussic acid are now. If any of our readers will turn to the fifth volume of the Medical and Physical Journal, page 201 *et seq.* and read the report of Hospital-Physician Magennis, respecting the treatment of seventy cases of phthisis in all its stages, they will be inclined to join us in scepticism, till the next twenty years shall have settled the anti-phthisical virtues of prussic acid.

We shall take a short case at random from Dr. Magennis.

"James Smith, aged 26, a seaman belonging to his Majesty's ship Ville de Paris, admitted on the 6th of October last. Has been ill a long time with phthisis; he was affected with every symptom which is the usual concomitant of the disease in its last and most aggravated stage; constant and deep-seated pain in both sides, but more particularly in the left; expectoration profuse, and rankly purulent, emitting a most disagreeable fœtor; regular attacks of febrile exacerbations every evening; profuse colliquative sweats; formerly subject to frequent hæmorrhages from the lungs, but nothing of that kind had occurred lately: He was reduced to the lowest state of debility, although twelve months before of Herculean powers, as one of his mess-mates informed me. The moment I saw this man, I pronounced it a lost case: Notwithstanding these unfavourable appearances, he began the Tinct. Digital. in small doses, which were gradually increased, and systematically persevered in till the 21st of November, on which day he was discharged, cured. The expectoration, originally a pint and a half, was reduced to about a table spoonful or less, and wholly free from purulence; the nightly perspirations had ceased upwards of twelve days; the thorax was completely freed from pain; and the cough had for some days totally disappeared, except for a few minutes after his first getting out of bed in the morning." *Med. and Phys. Journ.* vol. v. p, 206.

For the various other cases, twenty-five in number, *recovered* from the last, or purulent stage of phthisis by digitalis, we must refer to the volume alluded to. Whoever has been in the habit of examining the disorganizations in the lungs of those who die of phthisis, and have seen the very small portions of sound organ which sustain life in the last stages, must utterly despair of reparation, in such stages, unless the miracles of Medea's cauldron should again be wrought on earth.

Greatly as we have exceeded our limits, we must yet dedicate another page to the tenth and last section in Dr. Granville's work, which treats on the mode of prescribing the prussic acid. The chemical qualities of the medicine, and the incompatibles, are described in our review of the first edition, vol. ii. p. 272, and therefore we need not repeat them here. We shall add, however, a page of formulæ from our author.

"No. 2.—℞. Misturæ amygdalarum f ℥ viii.

Acidi Hydrocyanici med. m. x.

Syrupi tolutani f ʒ j.

M. *Cochleare unum maximum sumendum est tertia quâque horâ.*

---

"No. 3.—℞. Misturæ camphoræ f ℥ vi.

Tincturæ digitalis f ʒ ss.

Acidi Hydrocyanici med. m. xii.

F. M. *Singulis cochlearibus, tertia, vel quarta quâque horâ, sumenda.*

---

"No. 4.—℞. Potassæ subcarbonatis gr. xv.

Cocci cacti gr. viij.

Acidi Hydrocyanici med. m. x.

Aquæ stillatæ f ℥ vj.

F. M. *Infantibus, per parva cochlearia mistura, dum urget tussis propinatur: adultis vero, cochleare unum misturæ amplum, ter, vel quater de die, exhibetur.*

"No. 6.—℞. Decocti cinchonæ lancifoliæ f ℥ ii.

Acidi Hydrocyanici med. m. iij.

Spiritûs juniperi comp. f ʒ ss.

F. *Haustus ter quotidæ sumendus.*

---

"No. 8.—℞. Sodæ carbonatis gr. xl.

Cocci cacti gr. v.

Aquæ stillatæ f ℥ vj.

*Terantur optime & solutioni, per chartam bibulam diligenter colatæ, addantur.*

Acidi Hydrocyanici med. m. xii.

Syrupi papaveris f ʒ iiss.

F. M. L. A. *Cujus cochlearia iii. media, cum succi limonis recentioris, opportune edulcorati, f ℥ ss. in impetu effervescentiæ, tertiis horis, hauriantur.*" P. 408-9.

We must now take leave of Dr. Granville's work. We trust, that in the few commentaries which we have made, Dr. Granville will not find any tincture of cavilling criticism or arrogant dogmatism. Our observations have been chiefly argumentative; and whenever our author shews us that we are wrong, we shall submit to superior reasoning. If we have been sparing of censure, we have been still more so of eulogium. Those are not always our sincerest friends, who are loudest in our praises. While we can make allowances for the partialities of an inventor or reviver of a favourite remedy, we think it the most friendly act to draw the author's attention *repeatedly* towards the fallacious nature of medical evidence, as proved by all historical records. At the same time, we are not among those who discourage the introduction of every *new* remedy as a dangerous innovation.

We are, however, clearly of opinion that the profession are under deep obligations to Dr. Granville, of whose talents we entertain a very high opinion, for perseveringly bringing a medicine before the public in this country, which promises to hold some rank among our potent medicinal preparations.

